# Near‐complete genome of SARS‐CoV‐2 Delta variant of concern identified in a symptomatic dog *(Canis lupus familiaris)* in Botswana

**DOI:** 10.1002/vms3.1152

**Published:** 2023-04-29

**Authors:** Wonderful T. Choga, Samantha L. Letsholo, Chandapiwa Marobela‐Raborokgwe, Irene Gobe, Mbatshi Mazwiduma, Dorcas Maruapula, John Rukwava, Mary Gorettie Binta, Boitumelo J. L Zuze, Legodile Koopile, Kedumetse Seru, Patience Motshosi, Ontlametse Thato Bareng, Botshelo Radibe, Pamela Smith‐Lawrence, Kutlo Macheke, Lesego Kuate‐Lere, Modisa S. Motswaledi, Mpaphi B. Mbulawa, Mogomotsi Matshaba, Kereng V. Masupu, Shahin Lockman, Roger Shapiro, Joseph Makhema, Mosepele Mosepele, Simani Gaseitsiwe, Sikhulile Moyo

**Affiliations:** ^1^ Research Laboratory Botswana Harvard AIDS Institute Partnership Gaborone Gaborone Botswana; ^2^ Division of Human Genetics Department of Pathology Faculty of Health Sciences University of Cape Town Cape Town South Africa; ^3^ Faculty of Health Sciences School of Allied Health Professionals University of Botswana Gaborone Botswana; ^4^ Botswana National Veterinary Laboratory Gaborone Botswana; ^5^ VetPro Consultants Gaborone Botswana; ^6^ Department of Biological Sciences University of Botswana Gaborone Botswana; ^7^ Kweneng Veterinary Clinic Gaborone Botswana; ^8^ Health Services Management Ministry of Health and Wellness Gaborone Botswana; ^9^ Presidential COVID‐19 Taskforce Gaborone Botswana; ^10^ Health Services Management National Health Laboratory Gaborone Botswana; ^11^ Botswana‐Baylor Children's Clinic Centre of Excellence Gaborone Botswana; ^12^ Department of Immunology and Infectious Diseases Harvard T.H. Chan School of Public Health Boston Massachusetts; ^13^ Faculty of Medicine Department of Internal Medicine University of Botswana Gaborone Botswana

**Keywords:** Africa, Botswana, COVID‐19, dog, next‐generation sequencing (NGS), SARS‐CoV‐2

## Abstract

We sought to investigate whether SARS‐CoV‐2 was present, and to perform full‐length genomic sequencing, in a 5‐year‐old male crossbreed dog from Gaborone, Botswana that presented overt clinical signs (flu‐like symptoms, dry hacking cough and mild dyspnoea). It was only sampled a posteriori, because three adult owners were diagnosed with SARS‐CoV‐2 infection.

Next‐generation sequencing based on Oxford Nanopore Technology (ONT) was performed on amplicons that were generated using a reverse transcriptase real‐time polymerase chain reaction (RT‐qPCR) of confirmed positive SARS‐CoV‐2 nasopharyngeal and buccal swabs, as well as a bronchoalveolar lavage with mean real cycle threshold (qCt) value of 36 based on the Nucleocapsid (N) gene. Descriptive comparisons to known sequences in Botswana and internationally were made using mutation profiling analysis and phylogenetic inferences. Human samples were not available.

A near‐full length SARS‐CoV‐2 genome (∼90% coverage) was successfully genotyped and classified under clade 20 O and Pango‐Lineage AY.43 (Pango v.4.0.6 PLEARN‐v1.3; 2022‐04‐21), which is a sublineage of the Delta variant of concern (VOC) (formerly called B.1.617.2, first detected in India). We did not identify novel mutations that may be used to distinguish SARS‐CoV‐2 isolates from the dog and humans. In addition to Spike (S) region mutation profiling, we performed phylogenetic analysis including 30 Delta sequences publicly available reference also isolated from dogs. In addition, we performed another exploratory analysis to investigate the phylogenetic relatedness of sequence isolated from dog with those from humans in Botswana (*n* = 1303) as of 31 March 2022 and of same sublineage. Expectedly, the sequence formed a cluster with Delta sublineages – AY.43, AY.116 and B.1.617.2 – circulating in same time frame.

This is the first documented report of human‐associated SARS‐CoV‐2 infection in a dog in Botswana. Although the direction of transmission remains unknown, this study further affirms the need for monitoring pets during different COVID‐19 waves for possible clinically relevant SARS‐CoV‐2 transmissions between species.

## Graphical abstract

We detected and sequenced for the first time in Botswana a near‐full length SARS‐CoV‐2 genome in a 5‐year‐old male crossbreed dog from Gaborone, Botswana that presented overt clinical signs (flu‐like symptoms, dry hacking cough and mild dyspnea). The genome was a sub‐lineage of the Delta variant of concern (VOC) clade 20O and Pango‐Lineage AY.43 (Pango v.4.0.6 PLEARN‐v1.3; 2022‐04‐21). Our data suggest that persons with COVID‐19 infection should minimize contact with animals during illness or convalescent period.

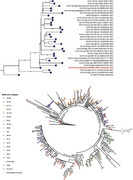

## 1 INTRODUCTION

Severe acute respiratory syndrome coronavirus 2 (SARS‐CoV‐2) is a zoonotic virus under genus betacoronavirus (β‐CoV) and the major aetiology of a pathological respiratory infection common in humans called coronavirus disease 2019 (COVID‐19) (Li et al., 2020). SARS‐CoV‐2 is postulated to have originated from either horseshoe bats (*Rhinolophus affinis*) or Malayan pangolins (*Manis javanica*), in China in 2019 (Andersen et al., 2020; Barua et al., 2021; Seyran et al., 2021; Sreenivasan et al., 2021). Strong support for this zoonotic origin is the fact that bat betacoronavirus was linked to the 2002−2003 SARS epidemic (SARS‐CoV), which was thought to be transmitted to humans either directly or through an intermediate host that is yet to be identified (Zhou et al., 2020; Zhou et al., 2020). Prior to the discovery of SARS‐CoV‐2, pets such as dogs could be infected with an Alphacoronavirus (α‐CoV), canine enteric coronavirus (CCoV), known to have mild symptoms and are not life‐threatening field (Decaro et al., 2021).

Recent evidence shows that anthroponotic infections of SARS‐CoV‐2 between animals and humans exists (Sit et al., 2020). The infection in dogs and cats is biologically feasible since they share the same cellular receptor, the angiotensin‐converting enzyme type 2 (ACE2) (Decaro & Lorusso, 2020; Decaro et al., 2021). Even though, dogs have little susceptibility to SARS‐CoV‐2, and the virus replicates poorly whereas cats and ferrets are more permissive to infection (Shi et al., 2020). Often, dogs that get infected with SARS‐CoV‐2 become asymptomatic with low viral ribonucleic acid (RNA) levels unless the RNA is isolated from nasal, oropharyngeal swabs and rectal swabs within 6 to 9 days post‐infection field (Barua et al., 2021; Shi et al., 2020; Sit et al., 2020). SARS‐CoV‐2 infection has also been reported in wild animals including minks, tigers, lions, and ferrets (Dalton et al., 2022; Decaro et al., 2021; Delahay et al., 2021; Mishra et al., 2021; Sharun et al., 2021). Reservoirs for SARS‐CoV‐2 including immunocompromised humans and animals are postulated to play a role in spurring the mutations of the virus that potentially leads to new variants

of concern in the evolving COVID‐19 pandemic; and different disease attributes (Otto et al., 2021; Shi et al., 2020). SARS‐CoV‐2 has been demonstrated to infect pets and most studies are not from African continent (Molini et al., 2022).

Although COVID‐19 has been declared a notifiable disease by the World Animal Health Organisation (OIE), it has not been stated as a notifiable disease in Botswana for animals. Due to this fact, there are no clear guidelines to assess nor manage animals infected with SARS‐CoV‐2 including pets, and on the management of those in close contact with infected humans. However, there has been lobbying by the Department of Veterinary Services (DVS) to other key stakeholders for the adoption of a One‐health approach to assessing human cases of COVID‐19, especially especially when wild or domestic animal–human close contact happens (Gibbs, 2014; WHO, O. 2017). Here we report the first case of SARS‐CoV‐2 in a dog from owners previously confirmed of being infected by SARS‐CoV‐2. The dog tested positive for SARS‐CoV‐2 reverse transcriptase real‐time polymerase chain reaction (RT‐qPCR) in nasopharyngeal swabs. The SARS‐CoV‐2 genome has been almost completely sequenced and clustered with the sequences that have been circulating among humans, especially from the dates of the dog case.

## 2 METHODS AND MATERIALS

### 2.1 Sample collection

This was part of SARS‐CoV‐2 genomic surveillance in response to the State of Emergency (SoE) in Botswana. During the time, the dog was swabbed; it presented COVID‐19‐like symptoms (including a dry hacking cough which worsened with exertion and mild depression) and, as a result, its owners had contacted the veterinary officials. The 5‐year‐old crossbreed male dog (*Canis lupus familiaris*) was living in a household with three adults who were under a 10‐day quarantine for a SARS‐CoV‐2 infection. The dog was at high risk of getting infected as it was being hand‐fed and cared for by everyone in the household during their quarantine.

After proper restraint by licensed veterinary personnel, without exerting undue discomfort to the dog (0.3 mL dexmedetomidine hydrochloride 0.5 mg/mL, 1.0 mL Ketamine 50 mg/mL, and 0.2 mL Butorphanol 10 mg/mL), bucal and nasopharyngeal swabs as well as bronchoalveolar lavage were obtained and tested for SARS‐CoV‐2 using the PCR method. Specimens were transported in 2–8°C and referred to the Botswana Harvard HIV Reference Laboratory (BHHRL) for SARS‐CoV‐2 testing and sequencing. At the time of testing, BHHRL had received ISO 15189 recognition by the Southern African Development Community Accreditation Services (SADCAS). BHHRL remains the main referral centre for SARS‐CoV‐2 testing genomic surveillance in Botswana.

### 2.2 DNA extraction and RT‐PCR

Nucleic acid extraction was carried out using a Nucleic Acid Extraction kit (Wuhan MGI Tech Co., Ltd, Wuhan, China) according to the manufacturer's instructions. For diagnostic real‐time polymerase chain reaction (PCR) analyses, the 2019‐nCoV RNA (PCR‐Fluorescence Probing) Assay (Sun Yat‐sen University, Da An Gene Co., Ltd, China) was used according to the manufacturer's instructions. An extraction control, master‐mix‐only control, positive control, and nontemplate control were included in the PCR run. To increase the yield of the RNA, the specimen was extracted twice, following centrifugation at 22,000 × *g* for 15 min. The resulting pellet was re‐suspended in lysis buffer before bead‐based automated extraction.

### 2.3 Tiling PCR, next‐generation sequencing and sequence analysis

Residual RNA from RT‐qPCR testing were retrieved for tiling PCR using Superscript IV (Invitrogen, Marseille, France) and the hexamer primers to produce complementary DNA (cDNA) (https://artic.network/ncov‐2019). Oligonucleotides (https://github.com/artic‐network/artic‐ncov2019/tree/master/primer_schemes/nCoV‐2019/V3) used were manufactured by Inqaba Biotech (Pretoria, South Africa). The amplicons were sequenced using MinION technology using Mk1B machine (Oxford Nanopore Technologies, Oxford, UK) running with MinKNOW Release 21.05.12. The generated FASTQ files were analysed using Genome detective (Cleemput et al., 2020; Vilsker et al., 2019). Prior to submitting the sequence in the Global initiative on sharing all influenza data (GISAID) database (https://www.gisaid.org) accession number EPI_ISL_8900015, the sequence quality and coverage were assessed using Nextclade v.1.7.4 (https://clades.nextstrain.org) (accessed 10‐11‐2021). At the time of this report, the lineage assignment was done using the Phylogenetic Assignment of named Global Outbreak LINeages (PANGOLIN version v.3.1.20 accessed on 2022‐02‐28) software suite (https://pangolin.cog‐uk.io/).

### 2.4 Sequence sorting and phylogenetic analysis

The downloaded FASTA files were analysed using NextClade to assess the coverage, lineage and quality. For reference sequences, we included all the near‐full length Delta VOC sequences from GISAD (*n* = 31 including from Botswana) at the time of the manuscript. Since there were few sequences, no further sampling strategies. The sequences were aligned using NextAlign, and the ensuing multiple sequence alignment (MSA) was used to construct phylogenetic tree. NC_044512 was used as a reference sequence. The tree topology was inferred by performing maximum likelihood (ML) analyses, and the bootstrap values were set at 1000, and the ModelTest v.3.7 (Posada, 2006) was used to select the simplest evolutionary model that adequately fit the sequence data. ML tree were implemented using FastTree (Price et al., 2009). Seaview tool was used to assess the architectures of the produced trees. The tree was visualised in FigTree v1.4.3 and posterior probabilities above 0.80 were noted as statistically significant.

## 3 RESULTS

In this report, we give details of the first detection, isolation and near‐full length genome sequencing of SARS‐CoV‐2 in an infected 5‐year‐old, crossbreed male dog in Gaborone, Botswana. A case of a dog with overt clinical signs, and living with three COVID‐19 infected humans was reported to Botswana National Veterinary Laboratory (BNVL) in Gaborone on the 3 August 2021. The dog showed presence of 4‐day history of a dry hacking cough by the time the report was made by the owner. Its owner and 2 other adult relatives had recently been diagnosed of COVID‐19 using the rapid antigen detection kit (lateral flow device (LFD)) on the 2 August 2021. The dog was a medium‐size dog with a body condition score of 2nd half out of 5. On clinical examination on the 5 August 2021, the dog was mildly depressed and had lost appetite. Efforts were made to rule out a kennel cough infection through clinical history, vaccine records and clinical examination. The dog had vaccination records (dated 27/04/2018 for Vanguard®Plus 5 and 17/08/2019 for Rabisin) and was protected from rabies but had probably waning protection for canine distemper, respiratory disease (canine adenovirus type 2 (CAV‐2), and canine parainfluenza), canine parvovirus, rabies, and infectious canine hepatitis (ICH) given the time lapse since vaccination. In‐contact neighbourhood dogs had no symptoms of the highly infectious kennel cough despite being in direct contact with the dog. This was the only symptomatic dog at the time of examination and in a week to two from the time of examination, making kennel cough a less likely diagnosis. The PCR test results were weakly positive for COVID‐19, with a qCt‐value of 36. Bronchoalveolar lavage and deep nasal swabs were taken on the 6 August 2021 and were found to be positive as well with Ct‐values of 38 and 36 respectively. The nucleic acid material was concentrated and sequenced using the MinION sequencer that is based on ONT technology.

**FIGURE 1:**
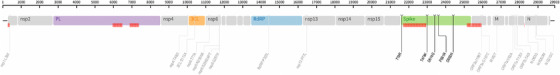
Full genome representation of a Delta SARS‐CoV‐2 sequence isolated from a dog, defining mutations with those in the Spike (green). Figure generated by covdb.stanford.edu.

We generated a near‐full length genome (∼90% coverage) that was classified as clade 20 O and Pango‐Lineage AY.94 (Pango v.3.1.20 2022‐02‐28), which is a sublineage a Delta variant of concern (VOC) (formerly called B.1.617.2, first detected in India). The mutation profile analysis of the sequence revealed several mutations including Spike (S) (T19R, T478K, D614G, P681R D950N), Membrane (N) (I82T), Nucleocapsid (N) (D63G, R203M, G215C), NS7a (T120I), NS7a (V82A), NS7b (T40I), NSP4 (T492I), NSP5 (S113A), NSP6 (Q257T), NSP6 (T77A), NSP12 (P323L), NSP13 (P77L) (Figure 1).

The Pango‐lineage assignment is mutations‐based and often dynamic (changes with update). Thus, we performed phylogenetic inferences based on maximum likelihood (ML). The dog was only sampled a posteriori, based on human test results but SARS‐CoV‐2 sequences isolated from owners were not available for analysis. Instead, we performed the ML phylogenetic analysis of Delta sequences (*n* = 1233) from Botswana (as of 21 April 2022) to investigate how the sequences isolated from dog cluster with others. Except for (*n* = 1) AY.94 isolated from the dog; all (*n* = 1303) sequences were isolated from humans. These were classified into 7 clades – G (*n* = 23), GH (*n* = 5), GK (*n* = 1219), GR (*n* = 37), GRA (*n* = 13), GV (*n* = 1), and O (*n* = 5) – and 32 sublineages of Delta including AY.1 (*n* = 9), AY.100 (*n* = 1), AY.105 (*n* = 1), AY.112 (*n* = 40), AY.116 (*n* = 326), AY.121 (*n* = 2), AY.122 (*n* = 11), AY.125 (*n* = 1), AY.127 (*n* = 2), AY.26 (*n* = 1), AY.36 (*n* = 1), AY.36.1 (*n* = 3), AY.39 (*n* = 4), AY.4 (*n* = 3), AY.4.2.3 (*n* = 2), AY.42 (*n* = 1), AY.43 (*n* = 16), AY.45 (*n* = 168), AY.46 (*n* = 664), AY.53 (*n* = 2), AY.54 (*n* = 1), AY.82 (*n* = 1), AY.91 (*n* = 14), AY.95 (*n* = 1), AY.99 (*n* = 4) and B.1.617.2 (*n* = 24).

## 4 DISCUSSIONS

To our knowledge, no previous SARS‐CoV‐2 RNA detection from a dog has been reported in Botswana or Southern Africa, and this is the first near‐full length nonhuman sequence to be generated from Africa. By the time of the manuscript, there were additional partial SARS‐CoV‐2 sequences from dogs from Egypt that were deposited in GISAID. This presence of SARS‐CoV‐2 RNA in dogs of this study has been described in dogs elsewhere (Sit et al., 2020) and the occurrence of SARS‐CoV‐2 has been linked to a number of factors, including the owner's contact (Jairak et al., 2022). The dog's owners sought veterinary care after noticing overt clinical indicators (flu‐like symptoms and weight loss), and the animal's COVID‐19 status was confirmed. Despite being unable to extract a human sequence, the discovered strain – AY.94, a sublineage SARS‐CoV‐2 Delta VOC (B.1.617.2) – was the most common, adding to the growing body of evidence for human to pet transmission. This is not the first study to report Delta variant (B.1.617.2) infection in dogs. Prior to this study, there were cases reported in Kansas, USA and Barcelona, Spain (Doerksen et al., 2021; Fernandez‐Bastit et al., 2021).

**FIGURE 2:**
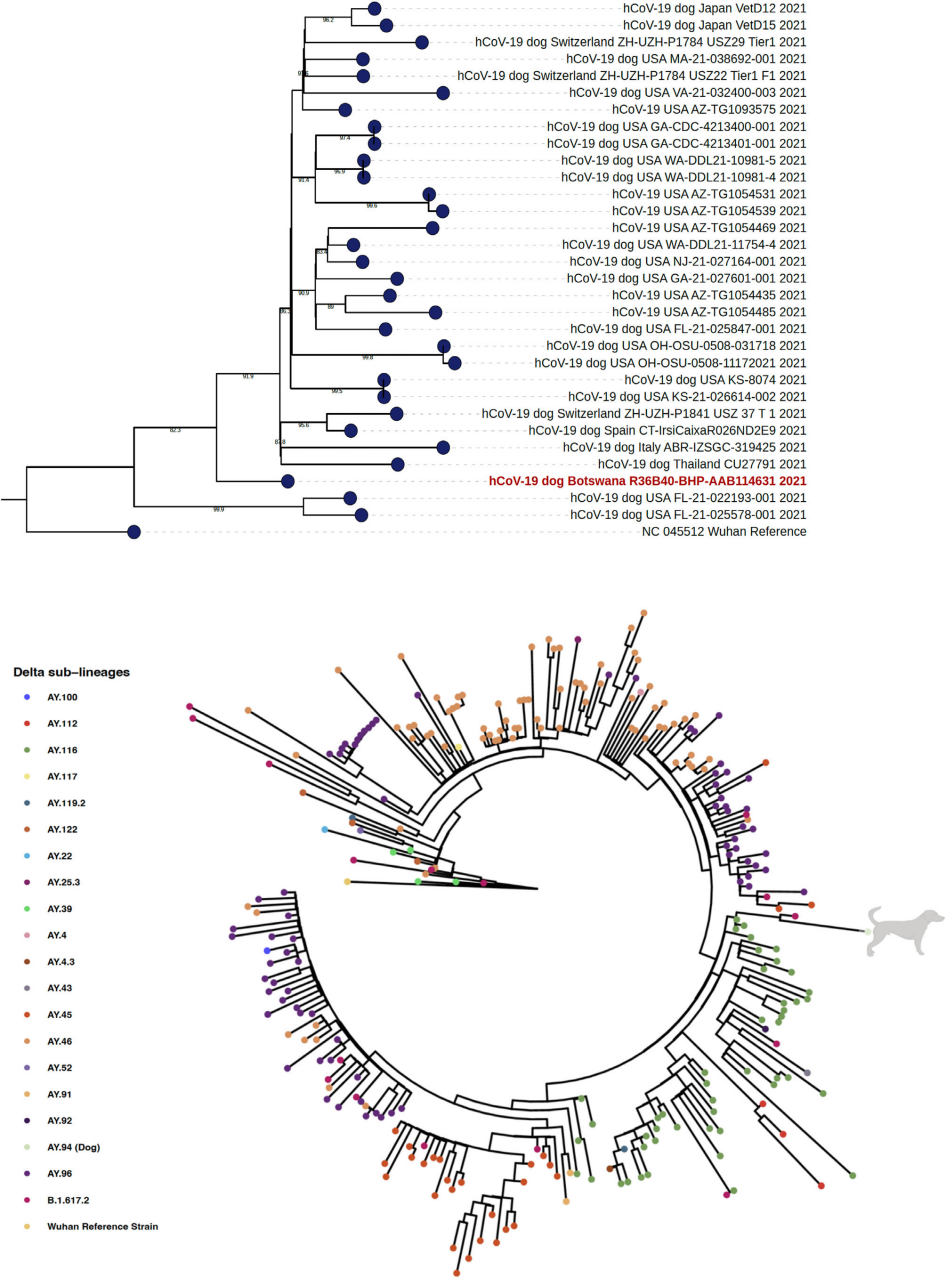
Phylogenetic tree showing the dog sequence with (top) 30 Delta sequences from dogs and (bottom) human Delta SARS‐CoV‐2 sequences isolated from Botswana circulating during the time the dog sample was swabbed.

Expectedly, phylogenetic analysis revealed that the viral genetic sequence from the dog was closely related to the Delta sublineage AY.43 cluster of humans that were in circulation in Botswana at the time of testing the dog Figure 2. Sublineage AY.34 was responsible for COVID‐19 in dog in dog in Spain (Fernandez‐Bastit et al., 2021). Although we speculate with caution that the transmission might have originated from humans, we do not disqualify other routes given evidence of relevant pet‐to‐human or pet‐to‐pet transmission were SARS‐CoV‐2 transmission dynamics in animal have been extensively investigate (Cui et al., 2022). Although we confirmed a positive SARS‐CoV‐2 infection in dog, signs were milder compared to what we have observed in symptomatic humans during the same epidemic wave. This corroborates previous studies showing pet infections as mostly asymptomatic or self‐resolving (Hamer et al., 2020).

We profiled the mutations in the Spike and all mutations agreed with those of Delta variant Delta variant of concern (B.1.617.2) across all the Spike domains. Some of the limitations of this study include the fact that phylogenetic analysis was performed only on the genome sequences of the dog and available human sequences as samples from SARS‐CoV‐2‐positive dog owners were not available due to the fact they were diagnosed using Rapid Antigen Tests. We presumed that the COVID‐19 infection from the dog was from the owners, who had COVID‐19 at the time. However, we cannot exclude the possibility that the dog acquired the infection from the environment including licking surfaces with SARS‐CoV‐2.

In conclusion, we present the first report of a nearly complete sequencing of the human‐associated SARS‐CoV‐2 in a dog in Botswana and Africa. Human‐to‐human transmissions rather than animal‐to‐human ones are what cause most reported SARS‐CoV‐2 outbreaks. However, our findings imply that people with COVID‐19 infection should limit contact with animals during illness or convalescent period since animals can be potential reservoirs of the virus. In addition, we recommend regular SARS‐CoV‐2 surveillance in domestic animals and increased awareness of VOC spillovers caused by high human–animal contact.

## AUTHOR CONTRIBUTIONS

Wonderful T. Choga: conceptualisation; data curation; formal analysis; methodology; visualisation; writing – original draft; writing – review & editing. Samantha L. Letsholo: conceptualisation; data curation; investigation; methodology; writing – review & editing. Chandapiwa Marobela‐Raborokgwe: supervision; writing – review & editing. Irene Gobe: supervision. Mbatshi Mazwiduma: investigation; writing – review & editing. Dorcas Maruapula: data curation; investigation; methodology; writing – review & editing. Mary Gorettie Binta: data curation; investigation; methodology; writing – review & editing. Legodile Koopile: investigation; methodology; writing – review & editing. Kedumetse Seru: investigation; methodology; writing – review & editing. Patience Motshosi: investigation; methodology; writing – review & editing. Ontlametse Thato Bareng: investigation; methodology; writing – review & editing. Botshelo Radibe: investigation; methodology; writing – review & editing. Kutlo Macheke: investigation; methodology; supervision; writing – review & editing. Lesego Kuate‐Lere: investigation; methodology; writing – review & editing. Modisa S. Motswaledi: investigation; methodology; supervision; writing – review & editing. Mpaphi B. Mbulawa: investigation; supervision; writing – review & editing. Mogomotsi Matshaba: investigation; methodology; supervision; writing – review & editing. Kereng V. Masupu: investigation; supervision; writing – review & editing. Shahin Lockman: investigation; project administration; supervision; writing – review & editing. Roger Shapiro: investigation; project administration; supervision; writing – review & editing. Joseph Makhema: project administration; supervision; writing – review & editing. Mosepele Mosepele: investigation; supervision; writing – review & editing. Simani Gaseitsiwe: conceptualisation; investigation; methodology; supervision; writing – review & editing. Sikhulile Moyo: conceptualisation; data curation; formal analysis; funding acquisition; investigation; methodology; project administration; supervision; writing – original draft; writing – review & editing.

## CONFLICT OF INTEREST STATEMENT

The authors declare no conflict of interest.

## FUNDING INFORMATION

European and Developing Countries Clinical Trials Partnership, Grant/Award Number: CSA2020NoE‐3104 TESAIII CSA2020NoE; Bill and Melinda Gates Foundation, Grant/Award Number: INV‐033558, INV‐036530; National Institute of Allergy and Infectious Diseases, Grant/Award Number: 5K24AI131924‐04, 5K24AI131928‐04; Fogarty International Center, Grant/Award Number: 3D43TW009610‐09S1; K43TW012350‐01; Foundation for Innovation in Diagnostics, Grant/Award Number: CV21‐0110; International Atomic Energy Agency, Grant/Award Number: INT0098

### ETHICS STATEMENT

Verbal consent was obtained from all dog owners after explaining the objectives and benefits of detecting and sequencing the viruses from the pet. Sequencing of SARS‐CoV‐2 is approved by the Health Research and Development Committee (HRDC).

### PEER REVIEW

The peer review history for this article is available at https://publons.com/publon/10.1002/vms3.1152.

## Data Availability

No.
